# Targeting of Rad51-dependent homologous recombination: implications for the radiation sensitivity of human lung cancer cell lines

**DOI:** 10.1038/sj.bjc.6602457

**Published:** 2005-03-22

**Authors:** A Sak, G Stueben, M Groneberg, W Böcker, M Stuschke

**Affiliations:** 1 Department of Radiotherapy, University Hospital Essen, 45122 Essen, Germany; 2 Institute of Medical Radiation Biology, University Hospital Essen, 45122 Essen, Germany

**Keywords:** Rad51, H2AX, p53, DNA double-strand break repair, apoptosis, lung cancer

## Abstract

The aim of the present work was to study the role of Rad51-dependent homologous recombination in the radiation response of non-small-cell lung cancer (NSCLC) cell lines. A dose- and time-dependent increase in the formation of Rad51 and *γ*-H2AX foci with a maximum at about 4 and 1 h after irradiation, followed by a decrease, has been found. The relative fraction of cells with persisting Rad51 foci was 20–30% in radioresistant and 60–80% in radiosensitive cell lines. In comparison, a higher fraction of residual Dsb was evident in cell lines with nonfunctional p53. Transfection with As-Rad51 significantly downregulates radiation-induced formation of Rad51 foci and increases apoptosis, but did not influence the rejoining of DNA double-strand breaks. Interestingly, wortmannin, a well-known inhibitor of nonhomologous end-joining, also inhibits Rad51 foci formation. In general, there was no correlation between the clonogenic survival at 2 Gy and the percentage of initial Rad51 or *γ*-H2AX foci after ionising radiation (IR). The most reliable predictive factor for radiosensitivity of NSCLC cell lines was the relative fraction of Rad51 foci remaining at 24 h after IR. Although most of the Rad51 foci are co-localised with *γ*-H2AX foci, no correlation of the relative fraction of persisting *γ*-H2AX foci and SF2 is evident.

Among the various forms of DNA damage induced by ionising radiation (IR), DNA double-strand breaks (Dsb) are the most lethal. Recognition and repair of Dsb is therefore a critical step in irradiated cells. In eukaryotes, DNA double-strand breaks (Dsb) can be repaired either by nonhomologous end-joining (NHEJ) mechanism or by homologous directed repair (Kanaar *et al*, 1998; Johnson and Jasin, 2001). An important repair protein responsible for NHEJ is DNA-dependent protein kinase (DNA-PK), a serine–threonine protein kinase consisting of three subunits (DNA-PKcs, Ku70 and Ku80). Cells with reduced DNA-PK activity due to a defect in any of the subunits are deficient in the rejoining of radiation-induced DNA Dsb and are radiosensitive in the clonogenic assay (Leuther *et al*, 1999; Smith and Jackson, 1999). On the other hand, Rad51 protein, a member of the Rad52 epistasis group, plays an essential role exclusively in homologous recombination (HR) in mammalian cells. The Rad51 protein is essential for the viability of mammals and vertebrates as mouse embryos and chicken cells with homozygous Rad51 deletion are not viable (Tsuzuki *et al*, 1996; Sonoda *et al*, 1998). Lethality of Rad51(−/−) mouse embryos was thought to be a consequence of p53-dependent apoptotic death of growing cells with accumulated spontaneous Dsb that are unrepaired by HR. Double mutants of Rad51(−/−) and p53(−/−) mice live longer due to the lack of p53-dependent apoptotic cell death. In general, the expression level of Rad51 correlates with the fraction of cycling cells (Shinohara *et al*, 1993; Yamamoto *et al*, 1996) and elevated levels of Rad51 protein was found in tumour cells as compared with normal cells (Raderschall *et al*, 2002a).

In human lymphocytes in the growing phase and in a low number of normal cultured mammalian cells, the Rad51 is detected in multiple nuclear foci, that is, focally concentrated immunofluorescence signals (Tashiro *et al*, 1996). These foci are thought to correspond to Rad51 containing nucleoprotein filaments in the Dsb recombination process (Haaf *et al*, 1995; Yamamoto *et al*, 1996) and contain the ssDNA-binding replication protein A (RPA), Rad52 and Rad54 (Golub *et al*, 1998; Gupta *et al*, 1998; Liu *et al*, 1999; Tan *et al*, 1999). Ionising-radiation-induced DNA damage increases the percentage of cells with Rad51 foci in a dose- and time-dependent manner, with a maximum at about 3–6 h after irradiation (Bishop *et al*, 1998). Therefore, a timely co-ordinated action of NHEJ and HR in mammalian cells is obviously, with a fast but error-prone (NHEJ) and a slow but error-free (HR) repair pathways (van Gent *et al*, 2001). A cell-cycle-dependent effect of Rad51 protein accumulation has been shown, with a peak in the S and G2 phases, in which the repair of Dsb primarily takes place via homologous recombination (Flygare *et al*, 1996). A number of approaches have shown that reduction in Rad51 levels via antisense oligonucleotides (Taki *et al*, 1996; Ohnishi *et al*, 1998), ribozymes (Collis *et al*, 2001) or drugs (Russell *et al*, 2003) increased the radiation sensitivity of tumour cell lines.

Given the role of DNA repair in the radioresponse of human tumour cells and the presence of two main repair pathways, we previously studied the role of DNA-PK-dependent NHEJ in the radiosensitivity of non-small-cell lung cancer (NSCLC) and have shown that reduced DNA-PK activity decreased the rejoining of radiation-induced DNA Dsb and increased the radiosensitivity (Sak *et al*, 2002). In the present study, the role of HR in the radioresponse of the same cell lines has been evaluated. We used As-oligodeoxynucleotide (ODN) specific for Rad51 mRNA in order to analyse its effect on Rad51-foci formation, rejoining of radiation induced DNA-Dsb and apoptosis in NSCLC cell lines. One of the earliest steps in the cellular response to DSBs is the phosphorylation of serine 139 of *γ*-H2AX. A quantitative similarity between the induction and repair of DSBs and the formation and disappearance of *γ*-H2AX foci has been found (Banath *et al*, 2004). Thus, we used *γ*-H2AX foci formation and a gel-electrophoresis assay to determine whether persisting Rad51 foci were indicative of non-repaired Dsb.

## MATERIALS AND METHODS

### Cell culture and irradiation of cells

The NSCLC cell lines H460, H520 and H661 were obtained from the American Type Culture Collection (ATCC, Rockville, MD, USA) and were grown in RPMI 1640 containing 10% foetal calf serum, and 100 U ml^−1^ penicillin/streptomycin under an atmosphere of 5% CO_2_, 95% air at 37°C. The NSCLC cell line A549 was obtained from DSZM (Braunschweig, Germany) and was grown in Eagle's minimal essential medium (MEM) supplemented with 15% foetal calf serum, non-essential amino acids and penicillin/streptomycin (100 U ml^−1^, all from Gibco-BRL, Paisley, UK). Cultures were irradiated with a ^60^Co source at 3.2 Gy min^−1^. Controls were mock irradiated. Measurement of clonogenic survival following irradiation of G1-enriched cells with 2 Gy (SF2), synchronisation of cells in the G1 and S/G2 phases of the cell cycle, and the fraction of apoptotic cells after staining with Hoechst-33342 (Sigma, Taufkirchen, Germany) was performed as previously described (Stuschke *et al*, 2002).

### Oligodeoxynucleotides

An antisense ODN targeting the Rad51 messenger RNA, one ODN with reverse orientation to Rad51 and one unrelated (UR) ODN were synthesised and purified by BioTez (Berlin, Germany). A BLASTN search of a database containing all sequences in the National Center for Biotechnology information (NCBI) database revealed no homology of the ODNs to other human genes. All oligonucleotides were phosphorothioates purified by high-pressure liquid chromatography. The sequences derived from a cDNA sequence (gene accession number U47077 from NCIB) were as follows: As-Rad51: 5′-CTG.CAT.CTG.CAT.TGC.CAT.TA-3′ (nucleotides: 231–250), gene accession number D14134 from NCIB; Sense-Rad51: 5′-TAA.TGG.CAA.TGC.AGA.TGC.AG-3′, Scramble-Rad51: 5′-GTC.TCA.GTC.CAT.TCT.ACT.A-3′. In order to control for unspecific effects of GG strings (Burgess *et al*, 1995) an UR sequence composition, 5′-AAG.AGA.GGT.CCG.AGG.AGG.GG-3′ (Iotsova and Stehelin, 1995) was also used. As-ODN targeting DNA-PKcs (Sak *et al*, 2002) was used in order to modulate NHEJ.

### Lipid-mediated transfection of antisense ODNs

Cells were plated in 9.6 cm^2^ culture dishes (Beckton Dickinson, Heidelberg, Germany) at a density of about 2 × 10^4^ cells cm^−2^ for H661 and 4 × 10^4^ cells cm^−2^ for the H460 and A549 cell lines. Transfections were carried out at 20 h after plating when cells reached a confluence of 50–80% as previously described (Sak *et al*, 2002).

### Immunofluorescence staining

#### Rad51

Cells were harvested and seeded in chamber slides and irradiated at 6 h for G1-enriched and at 24 h for exponential growing populations. Transfected cells were harvested at 20 h after the beginning of transfection, seeded in chamber slides and irradiated 6 h later. At specified times after irradiation, cells were fixed in 4% paraformaldehyde for 15 min at room temperature (RT), washed with PBS, followed by treatment in P-buffer (100 mM Tris-HCl, pH 7.4; 50 mM EDTA, 0.5% Triton X-100) for 15 min at RT, and washed twice in PBS. After incubation in blocking buffer (3% BSA, 0.1% Tween 20, 4 × SSC) for at least 24 h at 4°C, the primary Rad51 (Ab-1, Oncogene Research Products, San Diego, CA, USA) at a dilution of 1 : 500 in PBS was added and incubated for 20 h at 4°C or 90 min at RT. After washing in PBS, cells were incubated with a Cy3-labelled secondary antibody (goat-anti-rabbit, at a dilution of 1 : 500 in PBS) for 90 min at RT and washed twice in PBS.

#### *γ*-H2AX

For the most parts, immunostaining procedure for *γ*-H2AX foci detection was the same as described for Rad51. Cells were incubated with *γ*-H2AX antibody (anti-phospho-histone H2AX (Ser139), Upstate, NY, USA) at a dilution of 1 : 100 in PBS for 20 h at 4°C. After washing in PBS, cells were incubated with Alexa 488-labelled secondary antibody (goat-anti-mouse, at a dilution of 1 : 300 in PBS) for 2 h at RT and washed twice in PBS. Cells were incubated in the dark with 4′,6-diamidino-2-phenylindole (DAPI, 0.6 *μ*g ml^−1^ in PBS) for 10 min and coverslips were mounted in immu-mount (Shandon, Pittsburgh, PA, USA).

For conventional immunofluorescence microscopy, the cells were examined with a Zeiss Axioskop fluorescence microscope (Wetzlar, Germany) equipped with a CCD camera CS15/4MCC (Kappa Messtechnik GmbH, Gleichen, Germany). Images were captured and imported into an image analysis software package Image C (Imtronic GmbH, Berlin, Germany). In some cases images were merged using Photoshop 7.0 software (Adobe Systems Inc, USA). For quantitative analysis, nuclei were analysed by eye during the imaging process. At least 100 cells were selected at random and were counted to calculate the percentages of cells containing >5 Rad51 foci per nuclei. Alternatively, fluorescent images were collected with a confocal laser-scanning microscope (Leica DM IRE2, Wetzlar, Germany). For the quantitative analysis of the number of *γ*-H2AX foci per nuclei, about 10–20 cells per experiment were scored. For the analysis of Rad51 and *γ*-H2AX foci co-localisation, about 10–20 random cells per experiment that were scored as containing Rad51 foci were counted, analysed for *γ*-H2AX co-localisation and the results from at least three different experiments were averaged.

### Induction and repair of DNA double-strand breaks with the FAR assay

Cells were irradiated with 30 Gy at 4°C 20–24 h following transfection and were serially sampled at 0, 1 and 4 h after incubation at 37°C and cast into plug moulds. Cell lysis and electrophoresis were performed as described previously (Stuschke *et al*, 2002). The fraction of DNA released from the plugs into the gel (FAR) as a measure for DNA-Dsb was calculated according to the formula FAR=(intensity in the lane)/(intensity in the lane+intensity in the well).

### Detection of mRNA by Northern blot analysis

At 20–24 h following transfection, cells were harvested mechanically and washed once with PBS. Cell lysis and electrophoresis were performed as previously described (Sak *et al*, 2002). A PCR-amplified 1.15 kbp Rad51 fragment (Xia *et al*, 1997) was the specific probe for Rad51. For the synthesis of this probe, primers P1 (5′-TAG.AGA.AGT.GGA.GCG.TAA.GC-3′) and P2 (5′-ACC.CAA.TGA.TTC.AGT.CTT.TG-3′) were used. As a control for loading error and overall mRNA transcription activity, a plasmid probe for the *β-actin* gene was used (Ambion). Signals for each lane with Rad51 were normalised to the *β*-actin signals of the same lane.

### Western blot analysis

Cell lysis and electrophoresis were performed as described previously (Sak *et al*, 2003). Briefly, aliquots of total cell lysates (40 *μ*g) were resolved in 4–12% precasted polyacrylamide–SDS gels, and subjected to Western blot analysis using Rad51 (Oncogene Research Products, San Diego, CA, USA), *β*-actin (sc-1616-R, Santa Cruz Biotechnology, CA, USA) antibodies. Bound antibodies were detected by incubation with horseradish peroxidase (HRP)-conjugated secondary antibody, followed by enhanced chemiluminescence (Amersham, Germany) and autoradiography.

### Data evaluation

All experiments were repeated at least three times, and the data are given as a mean±standard error margin (s.e.m.) for the independent experiments. Statistical analysis (two-sided *t*-test) and graphs were performed with the aid of Microcal Origin version 7.0 (Microcal Software, Northampton, MA, USA).

## RESULTS

### Expression and modulation of repair complexes containing locally concentrated Rad51 foci

**Figure 1:**
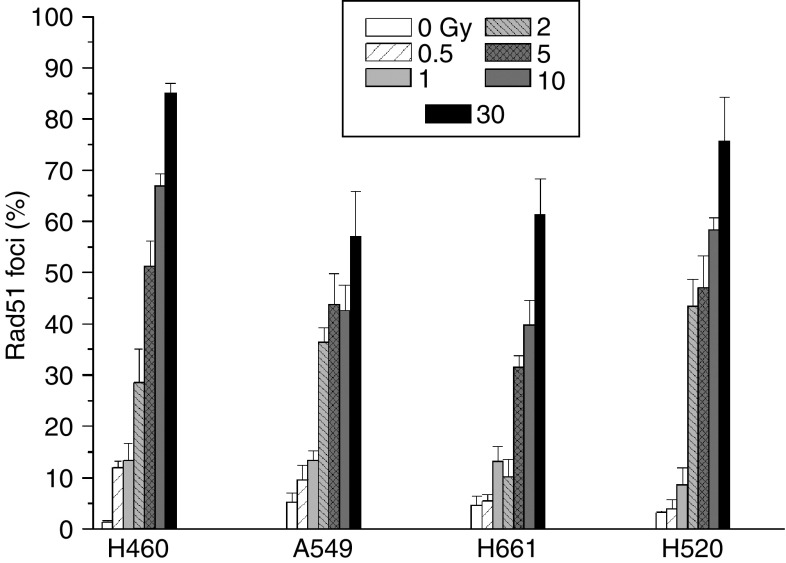
Radiation-induced Rad51 foci in NSCLC cell lines. Exponentially growing cells were exposed to IR (0, 0.5, 1, 2, 5, 10 and 30 Gy) and fixed for foci evaluation 4 h later. The respective mean values±s.e.m. of at least three independent experiments are shown.

**Figure 2:**
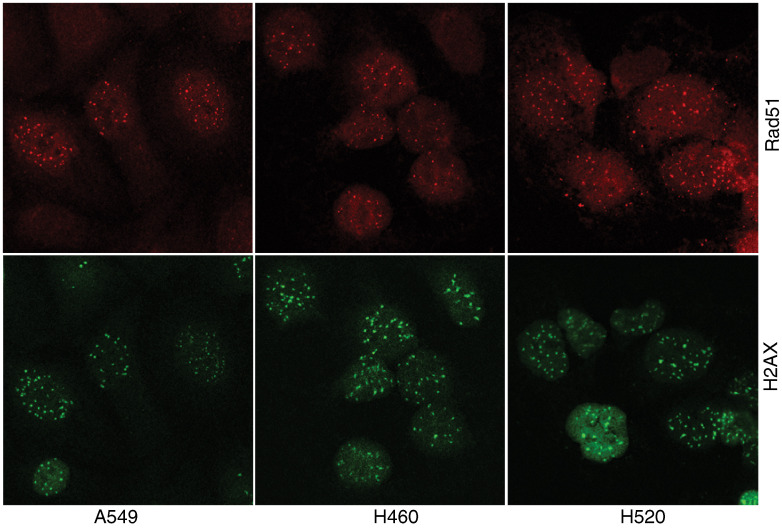
Radiation-induced Rad51 and *γ*-H2AX foci in NSCLC cell lines. Representative immunofluorescence micrographs of Rad51 (red, Cy-3) and *γ*-H2AX (green, Alexa-488) are shown at 24 h after 10 Gy.

The ability for the formation of Rad51 foci has been evaluated in exponentially growing and G1-enriched NSCLC cell lines with functional (A549, H460) and nonfunctional p53 (H661, H520). In exponentially growing populations, only a small percentage of nonirradiated cells with 1.7±0.4, 8.9±1.5, 5.2±2.5 and 6.2±2.6% of H460, A549, H661 and H520, respectively, showed more than five Rad51 protein foci per nucleus. In comparison, the percentage of cells with focally concentrated Rad51 foci increases in a dose- and time-dependent manner after IR, with a maximum at about 4 h. Figure 1 shows the percentage of Rad51-presenting nuclei at 4 h after irradiation with 0–30 Gy. The respective values are 85.0±2.0, 57.0±8.8, 61.2±7.1 and 75.6±8.7% for H460, A549, H661 and H520 cells irradiated with 30 Gy. The fraction of nuclei with Rad51 foci and the number of foci per nuclei increases with increasing radiation doses. In contrast to exponentially growing cells, the fraction of Rad51 foci in G1-enriched populations was very low with ⩽1% (A549, H460) and ⩽5% (H661, H520) without a significant effect of IR. At 24 h after irradiation, the number of foci per nucleus and the fraction of cells with Rad51 foci decreases (Figure 2, Table 1), showing that repair has taken place within this time interval. However, there was a difference in the fraction of Rad51 foci presenting cells at 24 h between the cell lines studied with <40% of the maximum level in IR resistant (A549, H661) and >60% in sensitive cell lines (H460, H520).

### Relation between Rad5 foci, *γ*-H2AX foci and residual DNA double-strand breaks

To determine whether persisting Rad51 foci at 24 h after irradiation were indicative of nonrepaired DNA double-strand breaks, *γ*-H2AX immunostaining and the FAR assay were used to evaluate the induction and repair of Dsb in NSCLC cell lines. The number of *γ*-H2AX foci continue to grow for about 1 h after irradiation and then decrease slowly over time, consistent with DNA double-strand break repair, but with slower kinetics, with a half-time of about 4 h. The initial number of *γ*-H2AX foci per nuclei after irradiation with 10 Gy was slightly higher for the cell lines with nonfunctional p53 (H661, H520) in comparison to the cell lines with functional p53 (A549, H460). The mean number of *γ*-H2AX foci per nuclei at 0 h after 10 Gy was 73.5±18.5, 88.5±11.5, 107.5±7.5 and 123.5±26.5 for H460, A549, H520 and H661 cells, respectively, whereby the numbers are possibly underestimated for H661 and H520. Both cells also showed high endogenous levels of *γ*-H2AX foci relative to A549 and H460. By 24 h after irradiation, the relative residual damage was significantly reduced in all cell lines studied (Figure 2, Table 1). Cell lines with nonfunctional p53 showed the most relative residual *γ*-H2AX foci in comparison to the cell lines with functional p53. In addition, residual DNA double-strand breaks have been measured with the FAR assay. There was no evidence for the presence of residual DNA strand breaks 24 h after irradiation with 30 Gy in the FAR assay. However, substantial amounts of residual damage were present for all cell lines 4 h after 30 Gy, with higher residual damage in H661 and H520, in comparison to the cell lines H460 and A549 (Table 1). The number of persisting *γ*-H2AX foci per nuclei at 24 h after irradiation with 10 Gy was by a factor of about 2 higher in comparison to the number of Rad51 foci. While 40–50% of the *γ*-H2AX foci were negative for Rad51, more than 80% of the Rad51 foci were also positive for *γ*-H2AX.

### Modulation of Rad51 expression and foci formation

**Figure 3:**
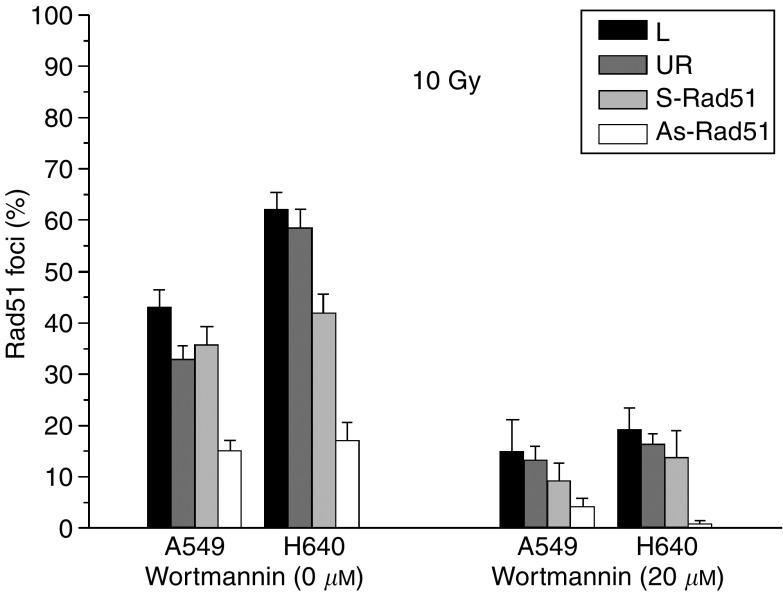
Effect of As-ODN and wortmannin on radiation-induced Rad51 foci in A549 and H460. Cells were transfected with lipofectamin only (L), UR ODN, sense- (S-Rad51) or antisense-ODN targeting Rad51 (As-Rad51), and treated with 20 *μ*M wortmannin (W20) or DMSO (W0) 1 h before irradiation with 10 Gy. Cells were fixed for foci evaluation at 4 h after irradiation.

Treatment of NSCLC cell lines with As-Rad51 resulted in a significant reduction of Rad51 foci in all cell lines and corresponds to a reduction in dose equivalents from 30 Gy to <2 (A549), ⩽5 (H460, H661) and ⩽10 Gy (H520). Interestingly, pretreatment of NSCLC cell lines with wortmannin, a well-known inhibitor of DNA-PK-dependent NHEJ pathway, increases Rad51 mRNA expression by a factor of about 1.6, but decreases the number of Rad51-positive cells by a factor of about 3 (Figure 3). These data show that radiation-induced formation of locally concentrated Rad51 foci, which are known to represent repair complexes of HR, is not attributable to the level of Rad51 expression and can be effectively inhibited not only with As-ODN targeting Rad51 but also with wortmannin.

**Figure 4:**
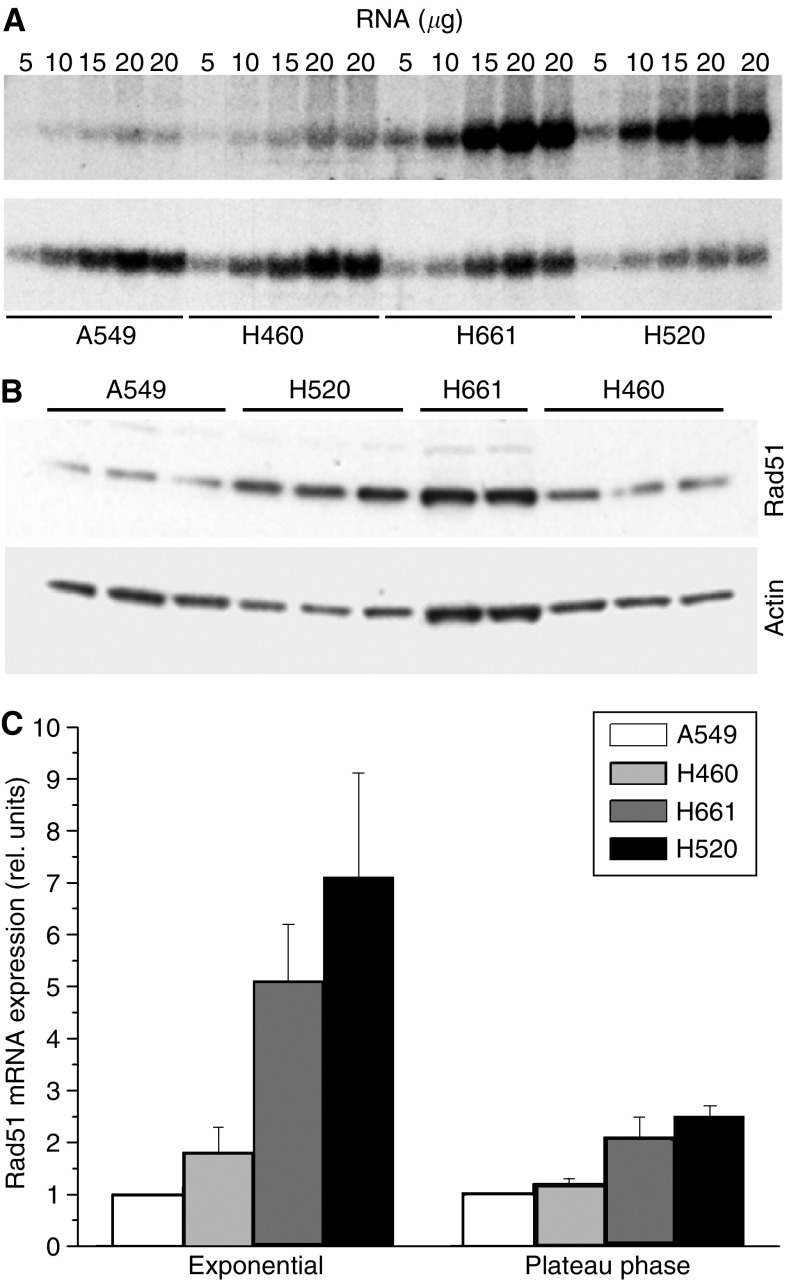
Representative hybridisation blots for the expression of Rad51 mRNA (**A**) and protein (**B**) in NSCLC cell lines are shown. Different amounts of total RNA (5–20 *μ*g) and protein (40 *μ*g) for each exponentially growing cell line were loaded onto the gel and hybridised with DNA probes or antibodies targeting Rad51 and *β*-actin. Data were normalised to the *β*-actin signal to correct for gel loading and differences in overall mRNA and protein expression. (**C**) Mean values±s.e.m. of at least three independent experiments for the relative level of Rad51 expression in NSCLC cell lines under exponential and plateau phase growth conditions are shown.

**Figure 5:**
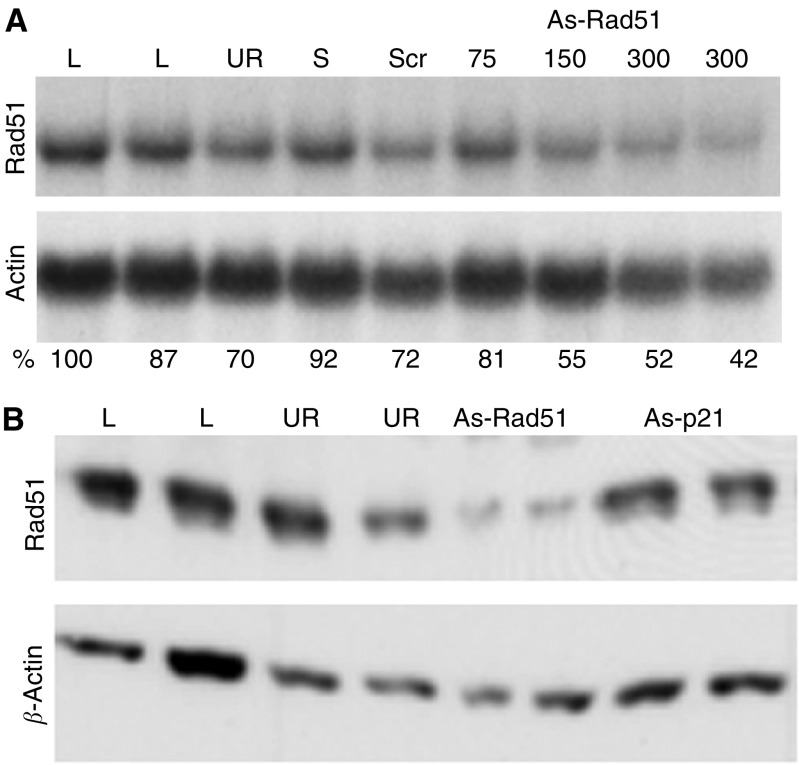
Representative hybridisation blots for the effect of As-Rad51 on the expression of Rad51 mRNA (**A**) and Rad51 protein (**B**) are shown. Cells were transfected for 24 h with lipofectamin only (L), UR ODN and As-ODN targeting Rad51 (As-rad51). As-p21 targeting p21^waf1/cip1^ (Sak *et al*, 2003) was used to show the specificity of the ODN used. Data were normalised to the *β*-actin signal to correct for gel loading and differences in overall gene expression.

To verify the specificity of the As-ODN, expression of Rad51 mRNA and protein was investigated after treatment with As-ODN. First, the basal expression level of Rad51 at the mRNA and protein levels in NSCLC cell lines was determined. As shown in Figure 4, the expression of Rad51 differed between the exponentially growing cell lines. Interestingly, both cell lines with nonfunctional p53, H661 and H520 in comparison to A549 and H460 with functional p53 show an increased expression of Rad51, both at the mRNA (5–7 folds) and protein (2–3 fold) levels. To test the hypothesis that As-ODN can initiate an RNAse H-mediated degradation of target mRNAs, we quantified the steady-state level of Rad51 mRNA in A549 and H460 cells after transfection with As-Rad51. As shown in Figure 5, treatment of H460 and A549 with 300 nM As-Rad51–3 results in a reduced Rad51 expression to about 65% with respect to lipofectamin controls.

### Effect of As-ODN on induction and rejoining of DNA-Dsb

**Figure 6:**
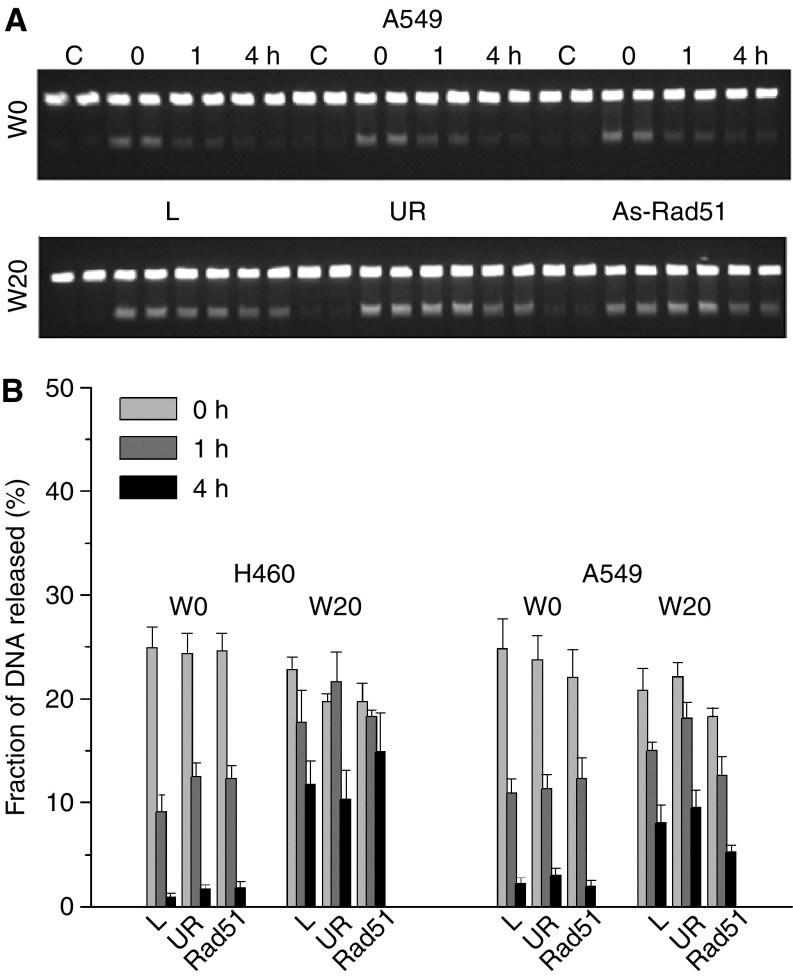
Rejoining of DNA-DSB in NSCLC cell lines treated with As-ODN. Constant-field gel electrophoresis showing the rejoining of IR-induced DNA-DSB in A549 cells after transfection with As-ODN (**A**). The fractions of DNA released into the gel as a measure for DNA Dsb at 0, 1 and 4 h after transfection and irradiation with 30 Gy are shown for A549 and H460 (**B**). Sham-irradiated controls (C) and irradiated cells previously transfected with lipofectamin only (L), UR ODN and As-ODN targeting Rad51 (As-rad51). Cells were treated with 20 *μ*M wortmannin (W20) or DMSO (W0) 1 h prior to irradiation with 30 Gy.

In order to outline the role of Rad51-dependent homologous recombination (HR) in the overall rejoining process of Dsb in NSCLC cell lines, rejoining kinetics were assessed in A549 and H460 over a 4 h period following exposure of cells to 30 Gy at 0 h (initial induction), 1 h (fast rejoining fraction) and 4 h (slow rejoining fraction) after transfection with As-ODN specific for Rad51. Figure 6A shows an example for Dsb rejoining of A549 cells after transfection with As-Rad51 and the respective results for both cell lines studied (Figure 6B). In both cell lines studied, no significant changes either in the initial (0 h) induction levels or in the fraction of Dsb rejoined after 1 and 4 h was found. These data show that HR has no measurable effect on radiation-induced Dsb rejoining in NSCLC cell lines. However, in order to exclude the possibility that the NHEJ pathway, which is the predominant one in human cells, may mask the role of HR in Dsb rejoining, cells were treated with wortmannin, a potent inhibitor of the NHEJ pathway, or As-ODN targeting DNA-PKcs. Treatment with 20 *μ*M wortmannin 1 h before irradiation with 30 Gy or As-DNA-PKcs increased the fraction of nonrepaired Dsb at 4 h after irradiation (Sak *et al*, 2002). If these nonrejoined Dsb are preferentially repaired via the HR pathway, combined pretreatment of cells with As-Rad51 and wortmannin or As-DNA-PKcs then should additionally increase the fraction of nonrejoined Dsb. However, combined pretreatment of cells with As-Rad51 and wortmannin or As-DNA-PK in comparison to the single-treatment schedule did not significantly influence the fractions of nonrejoined Dsb, either at 1 or at 4 h after irradiation. In conclusion, these data show that treatment of NSCLC cell lines with antisense ODN targeting Rad51 mRNA was not effective with respect to rejoining of radiation-induced Dsb, irrespective of whether the DNA-PKcs-mediated NHEJ pathway was active or not.

### Role of Rad51-dependent HR for the radiosensitivity of NSCLC cell

**Figure 7:**
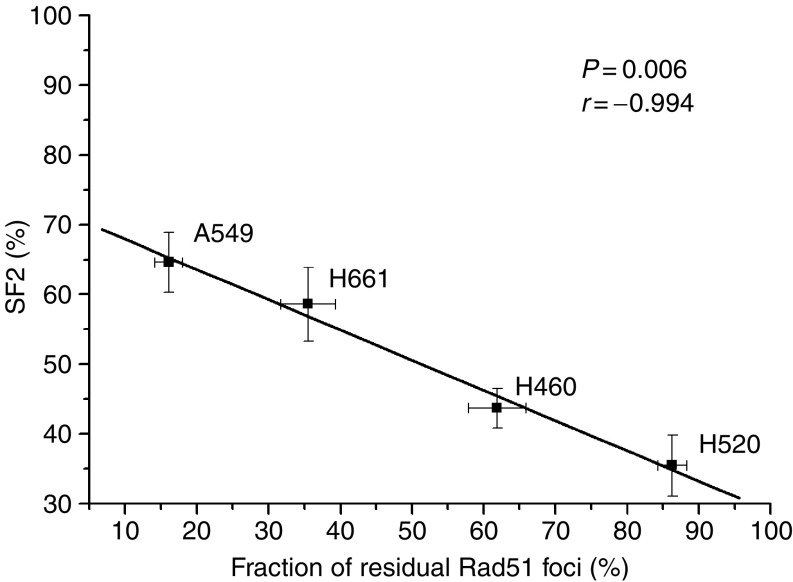
Relationship between the fraction of residual Rad51 foci remaining at 24 h (relative to the maximum level of Rad51 foci at 4 h) after irradiation with 10 Gy and clonogenic survival at 2 Gy (SF2) in NSCLC cell lines. A linear fit through the data points indicates an inverse relation between the fraction of remaining Rad51 foci and SF2 (*P*=0.006; *r*=−0.994).

In order to examine the relation between the Rad51-dependent repair activity, that is, formation of Rad51-containing repair complexes and survival, the fraction of apoptotic cells after a radiation dose of 20 Gy and clonogenic survival at 2 Gy (SF2) were determined. To evaluate the role of HR in the radiosensitivity of NSCLC cell lines, expression level of Rad51, fraction of Rad51 foci after irradiation as a functional test for Rad51 and the SF2 data have been compared in all the four NSCLC cell lines. Overall, there was no correlation between the SF2, as a measure for the intrinsic radiosensitivity, and the level of Rad51 expression or the fraction of Rad51 foci formation at 4 h after irradiation. Likewise, there was no correlation between SF2 and the fraction of residual *γ*-H2AX measured as the ratio of *γ*-H2AX foci at 0 and 24 h after irradiation with 10 Gy. However, the relative fraction of Rad51 foci remaining at 24 h (percentage of Rad51 foci at 24 h/percentage of Rad51 at 4 h) is a more reliable factor for the radiosensitivity of NSCLC cell lines (Figure 7).

**Figure 8:**
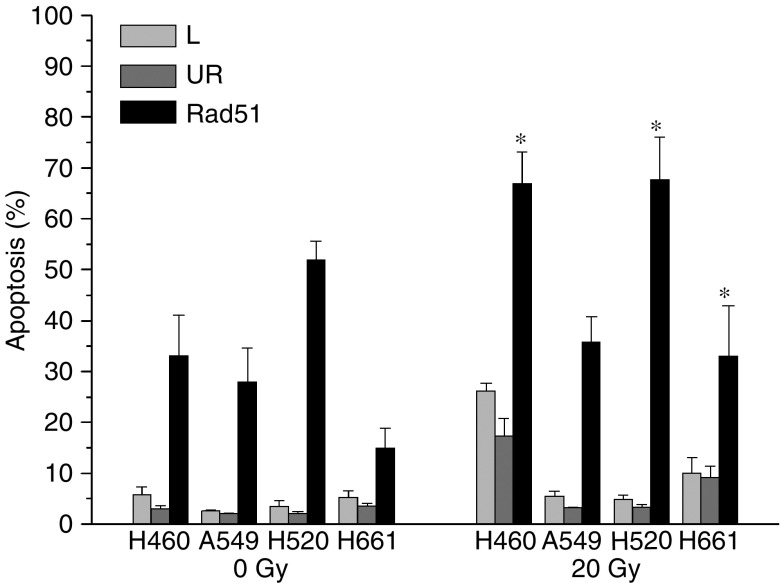
Radiation-induced apoptosis in NSCLC cell lines at 24 h following 20 Gy and transfection with lipofectamin (L), 300 nM UR and antisense Rad51 (Rad51) oligonucleotides, with a significant (^*^) difference between irradiated and nonirradiated cells (<0.05, *t*-test).

Even though the basal expression level and the percentage of Rad51 at early times after IR did not determine the radiosensitivity of NSCLC cell lines, the effect of decreased expression of Rad51 within an individual cell line may be more detrimental. In order to measure the effect of a selective Rad51 modulation within a given cell line, As-Rad51 were used. As shown in Figure 8, transfection with 300 nM As-Rad51 increased the fraction of apoptotic cells in all cell lines after irradiation with 20 Gy. However, increased apoptotic death of sham-irradiated cells was also evident. Even so, there is a significant increase of radiation-induced apoptosis in cells treated with As-Rad51.

## DISCUSSION

Repair of radiation-induced DNA double-strand breaks in mammalian cells has been thought to process mainly through the NHEJ, with DNA-PK being the central repair protein complex for this pathway. Nevertheless, HR, the most important repair pathway for Dsb in yeast, also contributes to the repair of Dsb in vertebrate cells (Sonoda *et al*, 2001) and is essential for their viability. Inactivation of the key HR protein Rad51 in chicken lymphocytes leads to an accumulation of cells in the G2/M with subsequent increase of chromosome aberrations prior to cell death (Sonoda *et al*, 1998). In the same way, murine cells deficient in Rad51 are nonviable (Tsuzuki *et al*, 1996). In comparison, cells overexpressing Rad51 showed higher recombination frequencies (Lambert and Lopez, 2001), reduced DNA break frequencies, translocations, chromosome aberrations, decreased induction of apoptosis (Grandy *et al*, 2002; Raderschall *et al*, 2002b), stimulated tumorigenesis (Bertrand *et al*, 2003) and increased resistance to IR (Vispe *et al*, 1998).

To evaluate the role of Rad51 in the radiosensitivity of NSCLC cell lines and the rejoining process of radiation-induced Dsb, expression of Rad51 was modulated with As-ODN targeting Rad51. Since complete loss of *Rad51* gene expression leads to cell death in vertebrate cells, the use of As-ODN represents a rational tool and an opportunity to analyse the impact of the modulation of Rad51-dependent homologous recombination pathway in the radiosensitivity of human NSCLC cell lines. After a moderate downregulation of Rad51 expression to about 60% of the nontransfected cells, the formation of Rad51 foci, overall rejoining of Dsb, induction of apoptosis and clonogenic survival were evaluated after exposure of NSCLC cell lines to IR. In addition, *γ*-H2AX foci formation as a measure for DNA double-strand breaks and its co-localisation with Rad51 were also evaluated.

The present data demonstrate a dose- and time-dependent increase in the percentage of cells with Rad51 foci in NSCLC cell lines. Cell lines with nonfunctional p53, in comparison to the cell lines with functional p53, show a higher expression level of Rad51 at the mRNA level by a factor of about 2 and 5 in G1- and G2-enriched populations, respectively. It has been previously shown that wild-type p53 protein downregulates the level of Rad51 expression in primary cells (Xia *et al*, 1997). Reduced expression of Rad51 and reduced spontaneous formation of Rad51 foci have also been found in p53^+/+^ in comparison to p53^−/−^ primary mouse embryonic fibroblasts (Kumari *et al*, 2004). The activity of Rad51 was shown to be regulated by a transcription-independent direct protein–protein interaction with p53 (Linke *et al*, 2003). In cases where p53 is absent or mutated, Rad51 has been shown to have an increased recombination activity. In the present study, however, the high expression level of Rad51 found in cell lines with nonfunctional p53 is not reflected by a high fraction of Rad51 foci-presenting cells. Instead, the proliferation status of tumour cell lines primarily determines the formation of Rad51 foci after IR. Higher expression of Rad51 by a factor of about 2 and a high fraction of Rad51 foci in proliferating (>40%), in comparison to G1-enriched, confluent populations (<5%) was also found in NSCLC cell lines, irrespective of the p53 status. A clear cell-cycle-dependent expression of Rad51 foci has also been found in previous studies with higher fraction of Rad51 foci-presenting cells in the S- and G2-phases in comparison to the G1-phase (Yamamoto *et al*, 1996; Yuan *et al*, 2003). The highest fraction of Rad51 foci was found at 4–6 h after irradiation, a time where most of the initially radiation-induced Dsb are rejoined via the DNA-PK dependent non-HR mechanism. After this time, the percentage of cells with Rad51 foci gradually decreases. A high fraction of cells with persisting Rad51 foci at 24 h after irradiation was found in radiosensitive cell lines. These persisting rad51 foci possibly reflect Dsb rejoining products sensing the recruitment of HR pathway, obviously with different efficacy in the NSCLC cell lines studied.

In order to explore the possibility that residual Rad51 foci reflect nonrepaired DNA double-strand breaks, phosphorylation of histone *γ*-H2AX in response to DNA double-strand breaks produced by IR has been measured, which is proposed to concentrate repair factors at sites of DNA damage (Celeste *et al*, 2003). Although differences in the loss of *γ*-H2AX foci have been found to be related in part to the intrinsic radiosensitivity of cervical cancer cell lines (Banath *et al*, 2004), our data did not support these observations. A co-localisation frequency of more than 80% for Rad51 and *γ*-H2AX foci elucidate that persisting Rad51 foci mostly contains DNA double-strand breaks. However, the Rad51-positive foci, in comparison to the H2AX-positive but Rad51-negative foci, obviously have more correlative power for the radiation sensitivity of lung cancer cell lines.

Treatment of exponentially growing cells with As-Rad51 significantly reduces the percentage of cells with Rad51 foci in all NSCLC cell lines, but did not change the fate of Dsb rejoining in the FAR assay. Therefore, no indication of an involvement of Rad51 in the direct rejoining process of radiation-induced Dsb is evident from these data. Even under the condition where the NHEJ pathway is disabled with wortmannin, As-Rad51 has no measurable effect on Dsb rejoining. However, at concentrations resulting in a significant inhibition of DNA-PK-dependent NHEJ, wortmannin also inhibits ATM (Sarkaria *et al*, 1998). It has been shown that ATM regulates other proteins known to play a role in DNA-repair, cell-cycle regulation and radiation sensitivity, including p53, CHK2, BRCA1 and NBS1 (Canman *et al*, 1998; Cortez *et al*, 1999), and is required for the assembly of Rad51 foci (Chen *et al*, 1999; Yuan *et al*, 2003). Therefore, wortmannin should not only affect NHEJ through inhibition of DNA-PK activity in NSCLC cell lines (Sak *et al*, 2002), but also HR via inhibition of ATM-dependent formation of Rad51 foci. Conflicting results regarding the effect of wortmannin on HR has been published, with an increase (Delacote *et al*, 2002) and a decrease of HR (Paull *et al*, 2000; Allen *et al*, 2003). Our results, however, show an efficient inhibition of Rad51 foci formation after treatment with wortmannin, which in part explains its higher effect on the clonogenic survival of NSCLC cell lines when compared to a treatment schedule using As-ODN targeting DNA-PKcs (Sak *et al*, 2002). Inhibition of Rad51 foci with wortmannin is not a result of reduced Rad51 expression, but is obviously regulated at the post-translational level, because treatment with wortmannin leads to an increased expression of Rad51 mRNA. The observed effect of wortmannin on NHEJ, HR and cell survival is obviously the result of noncompetitive and covalent binding of wortmannin to the kinase region of DNA-PKcs protein (Izzard *et al*, 1999). The formation of covalent adducts may therefore prevent the dissociation of DNA-PKcs from the DNA ends, and thereby block both HR and NHEJ. Alternatively, but not exclusively, wortmannin may inhibit HR through inactivation of ATM, and thereby prevents Rad51 foci formation. Nevertheless, a selective inactivation of NHEJ via As-ODN targeting DNA-PKcs, the key repair protein of NHEJ, also did not change the Dsb rejoining response of NSCLC cell lines to As-Rad51 treatment. Therefore, no contribution of Rad51-dependent HR in the direct removal of radiation-induced Dsb as measured by the FAR assay is evident from these data. In this respect, Wang *et al* (2001) also have shown that HR did not measurably contribute to the removal of radiation-induced Dsb, even under conditions where NHEJ is compromised.

Despite the obvious missing of an involvement of Rad51 in the direct rejoining process of radiation-induced Dsb, loss of Rad51 has a deleterious effect on cell survival (Tsuzuki *et al*, 1996; Sonoda *et al*, 1998). In the same manner, downregulation of Rad51 foci formation has been shown to increase apoptotic cell death (Raderschall *et al*, 2002b). Our data support these observations and show that treatment of NSCLC cell lines with As-Rad51 increases radiation-induced apoptosis. Interestingly, the most apoptosis-proficient cell lines after As-Rad51 treatment are H520 and H460, both cell lines with high Rad51 foci remaining at 24 h after irradiation.

Even though Rad51 is expressed at higher levels in tumour cells as compared with normal cells (Raderschall *et al*, 2002a), modulation of Rad51 expression has been shown to be more susceptible in tumour cells (Russell *et al*, 2003). The increased expression of Rad51 found in tumour cells can be partially attributed to the higher proliferating fraction of tumour cells, because a higher expression of Rad51 mRNA by a factor of about 2 in exponentially growing, in comparison to confluent, NSCLC cell lines is evident from the present study. Nevertheless, the different regulatory processes of Rad51 in normal and tumour cells (Russell *et al*, 2003) and its role in radiosensitivity offer the possibility for a selective targeting of radiotherapy.

In conclusion, these data demonstrate that human NSCL cell lines have the same percentage of cells with Rad51 foci at early times after IR, albeit the significant differences in Rad51 expression. Therefore, differences in Rad51 expression alone did not determine the proficiency of Rad51 foci formation. Instead, cell cycle distributions at the irradiation time primarily affect the formation of IR-induced Rad51 foci. Downregulation of Rad51 expression within a specific cell line decreases the fraction of Rad51 foci-presenting cells and significantly increases radiation-induced apoptotic cell death. The relative level of persisting Rad51 foci measured 24 h after irradiation neither correlates to the relative level of persisting *γ*-H2AX foci nor to the residual level of DNA double-strand breaks, but was significantly correlated with SF2.

## Figures and Tables

**Table 1:** Comparison of residual damage and clonogenic survival in NSCLC cell lines

	**A549**	**H460**	**H520**	**H661**
Fraction of residual Dsb (%)^a^	8.0±0.3	10.0±0.4	20.0±6.0	22.0±1.3
Fraction of residual H2AX foci (%)^b^	19.0±1.7	19.0±4.2	38.0±5.5	67.0±3.0
Fraction of residual Rad51 foci (%)^c^	16.1±1.9	61.9±4.0	86.3±2.0	35.5±3.8
Clonogenic survival at 2 Gy^d^	64.6±4.3	43.7±2.8	35.5±4.4	58.6±5.3

a Fraction of residual Dsb (4 h/0 h) after a irradiation dose of 30 Gy, as measured with the FAR assay.

b Fraction of residual *γ*-H2AX foci (24 h/0 h) after irradiation with 10 Gy, as measured by *γ*-H2AX immunfluorescence.

c Fraction of cells with persisting Rad51 foci (24 h/4 h) after irradiation with 10 Gy, as measured by Rad51-immunfluorescence.

d Fraction of clonogenic survival after irradiation with 2 Gy.
